# Precision Nanometrology: Laser Interferometer, Grating Interferometer and Time Grating Sensor

**DOI:** 10.3390/s25216791

**Published:** 2025-11-06

**Authors:** Can Cui, Xinghui Li

**Affiliations:** Institute of Data and Information (iDI), Tsinghua Shenzhen International Graduate School (SIGS), Tsinghua University, Shenzhen 518055, China; cuic23@mails.tsinghua.edu.cn

**Keywords:** laser interferometer, grating interferometer, time grating sensor, precision nanometrology, manufacturing

## Abstract

Displacement metrology with nanometer-level precision over macroscopic ranges is a key foundation for modern science and engineering. This review provides a comparative overview of Precision Nanometrology, covering measurement ranges from micrometers to meters and accuracies between 0.1 nm and 100 nm. Three main technologies are discussed: the Laser Interferometer (LI), the Grating Interferometer (GI), and the Time Grating Sensor (TGS). The LI is widely regarded as the traceable benchmark for highest resolution; the GI has been developed into a compact and stable solution based on diffraction gratings; and the TGS has emerged as a new approach that converts spatial displacement into the time domain, offering strong resilience to environmental fluctuations. For each technique, the principles, recent progress, and representative systems from the past two decades are reviewed. Particular attention is given to the trade-offs between resolution, robustness, and scalability, which are decisive for practical deployment. The review concludes with a comparative analysis of performance indicators and a perspective on future directions, highlighting hybrid architectures and application-driven requirements in precision manufacturing and advanced instrumentation.

## 1. Introduction

**Figure 1:**
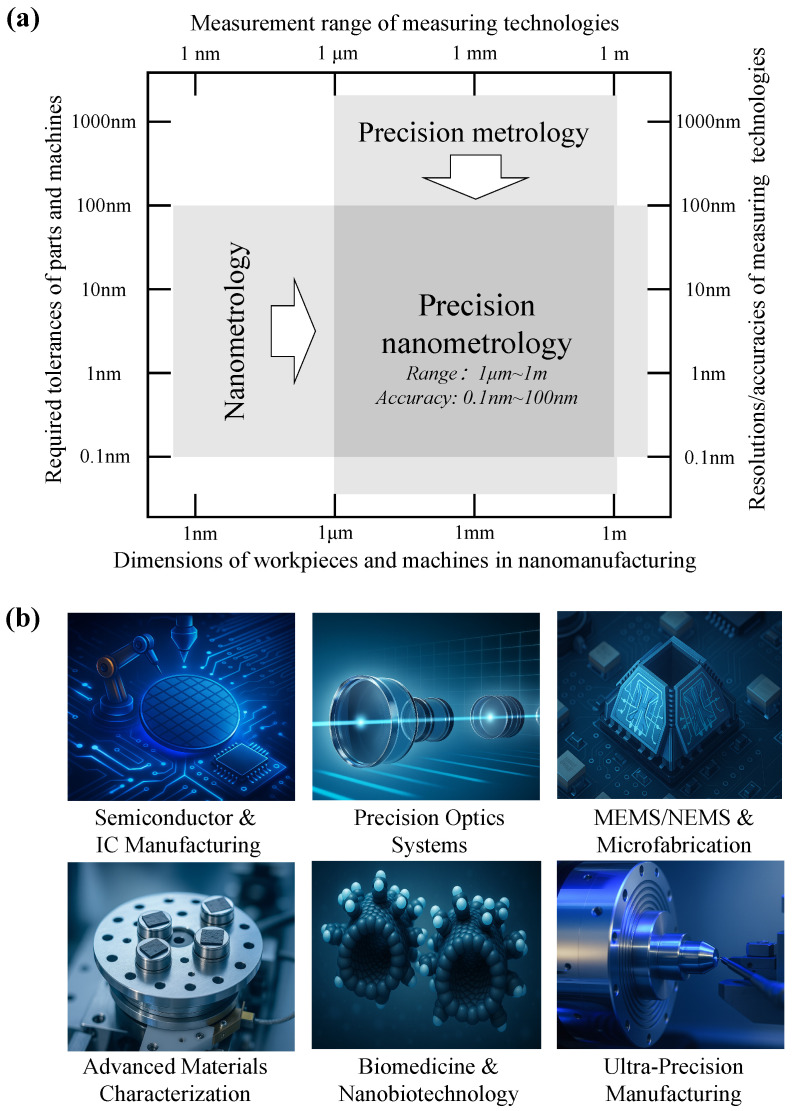
(**a**) Definition of the field of Precision Nanometrology [13,14]. (**b**) Application fields of Precision Nanometrology technology [24,29,36,37,38,39].

The ongoing pursuit of miniaturization and higher performance across science and industry has made precision displacement measurement a core enabling technology [1,2,3,4,5,6,7,8]. In fields ranging from fundamental physics to large-scale manufacturing, nanometer-level control and verification of position and motion are now basic requirements rather than specialized needs [9,10,11,12]. This has led to the formation of a research field often termed Precision Nanometrology [13,14,15,16,17,18,19,20,21,22,23], which addresses measurements from the micrometer to the meter scale with accuracies between 0.1 nm and 100 nm, as shown in Figure 1a. This discipline connects atomic-scale studies with macroscopic engineering, providing the metrological basis for emerging technologies.

The influence of Precision Nanometrology spans many advanced sectors, as illustrated in Figure 1b. In Semiconductor & IC Manufacturing [24,25,26,27,28], it is essential for wafer stage positioning, overlay control, and mask alignment, where accuracy directly determines yield and device performance. For Precision Optical Systems [29,30,31,32,33,34,35], such as telescopes and satellite imaging, it ensures surface quality and alignment stability. In MEMS/NEMS & Microfabrication [36], it supports process monitoring and device validation. In Advanced Materials Characterization [37], it enables quantitative evaluation of mechanical and thermal properties, such as nanoindentation and thermal expansion. In Biomedicine & Nanobiotechnology [38], it allows manipulation of biomolecules and study of cell mechanics. In Ultra-Precision Manufacturing [39], it provides dimensional control for aerospace, defense, and healthcare components with sub-micrometer tolerances. In addition, large-scale scientific facilities, including synchrotrons, particle accelerators, and gravitational wave detectors, rely increasingly on nanometrology to guarantee the stability and fidelity of their measurement infrastructure. Progress in these areas is tightly linked to advances in metrology. Looking forward, precision nanometrology is anticipated to advance toward tighter integration with artificial intelligence for intelligent data processing, the development of cross-scale metrology frameworks that unify nano-to-macro measurements, and to achieve unprecedented accuracy and long-term stability.

To meet the demanding requirements of these applications, several displacement measurement technologies have been investigated. Previous studies indicate that alternative displacement sensing methods have also been explored. Capacitive sensors are commonly employed in precision positioning systems due to their simplicity and high resolution at short ranges; however, they are strongly influenced by environmental factors such as humidity and temperature, and their usable range is typically limited to the sub-millimeter scale, which restricts their role in nanometrology applications. Optical frequency comb–based displacement metrology provides outstanding accuracy and absolute referencing capabilities, but it requires complex stabilization, calibration, and bulky optical setups, which hinder its integration into compact industrial instruments. These limitations explain why capacitive and frequency-comb methods, while promising, are less suitable than interferometric and grating-based approaches for the stringent requirements of precision nanometrology. Nevertheless, their continued development highlights the diversity of strategies being pursued to extend the limits of displacement sensing.

Among the available approaches, three techniques have become the main pillars of Precision Nanometrology: the Laser Interferometer [40], the Grating Interferometer [41], and the Time Grating Sensor [42]. Laser interferometers, traceable to the international definition of the meter through the laser wavelength, offering sub-nanometer resolution with well-established optical and electronic signal processing. Grating interferometers, which use diffraction gratings as the reference, provide advantages such as reduced sensitivity to environmental fluctuations and simpler alignment. The Time Grating Sensor, a newer concept, encodes displacement as a temporal phase variation and thus offers an alternative to conventional interferometric schemes. Its conceptual novelty has attracted increasing attention, although practical implementations are still in their early stages compared with interferometer-based systems.

**Figure 2:**
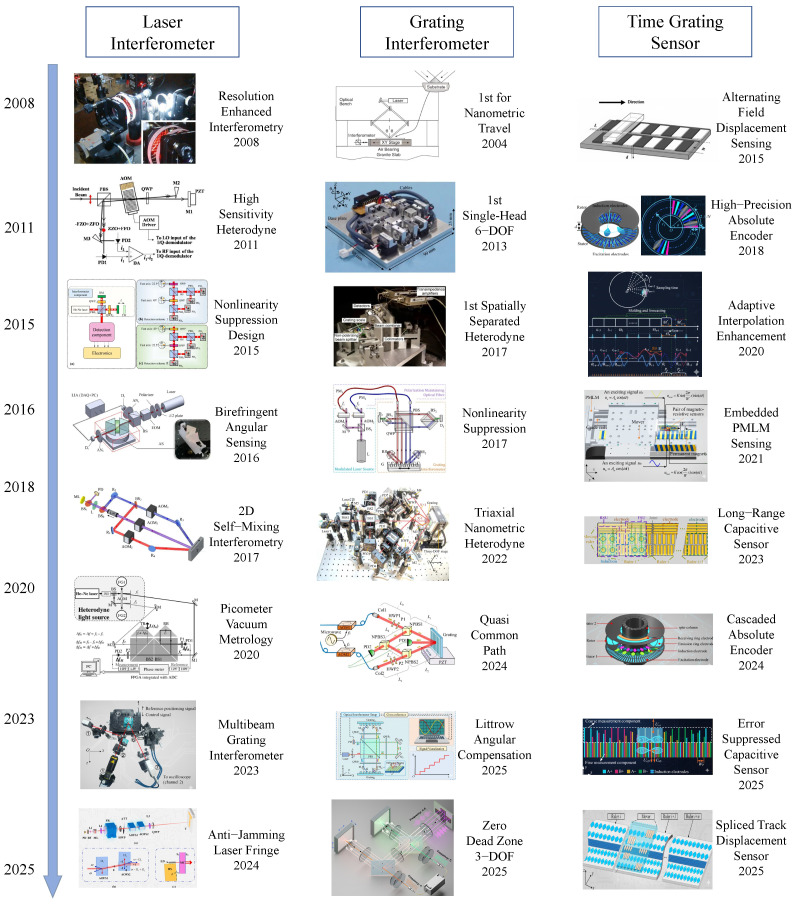
Remarkable Progress and Key Innovations in Nanometrology. This figure serves as a visual compilation showcasing the chronological evolution and significant breakthroughs in (1) Laser Interferometers (LI) (copyright Optica/IEEE/Elsevier/IOP/Wiley, reproduced with permission from [43,44,45,46,47,48,49,50]), (2) Grating Interferometers (GI) (copyright Optica/IEEE/Elsevier/IOP/Wiley, reproduced with permission from [41,51,52,53,54,55,56,57]), and (3) Time Grating Sensors (TGS) (copyright Optica/IEEE/Elsevier/IOP/Wiley, reproduced with permission from [42,58,59,60,61,62,63,64]) over the past two decades. Each column highlights representative hardware advancements and conceptual innovations that have pushed the boundaries of precision nanometrology.

The past two decades have brought major progress in all three technologies, with continued efforts to improve performance, integration, and versatility, as summarized in Figure 2. For laser interferometers, advances include the development of multi-axis systems capable of tracking up to six degrees of freedom, refined environmental compensation methods to reduce air refractive index effects, and the use of femtosecond optical frequency combs for absolute distance measurement. For grating interferometers, research has focused on miniaturizing sensor heads, designing two-dimensional grating standards, and embedding encoders into compact precision positioning platforms. Time grating sensors have progressed quickly from early proof-of-concept setups to systems reaching nanometer and even sub-nanometer resolution, with efforts directed toward improving linearity, extending measurement range, and enhancing resistance to electromagnetic interference. Despite these advances, the three methods exhibit different balances between accuracy, robustness, and scalability, which motivates a systematic comparative review.

This article provides a comparative survey of the three core approaches in Precision Nanometrology. Section 2 introduces key performance metrics for benchmarking displacement sensors. Section 3 outlines the fundamental operating principles of each technique. Section 4, Section 5 and Section 6 review recent progress, main innovations, and representative systems for laser interferometers, grating interferometers, and time grating sensors. Where appropriate, emphasis is placed on cross-comparison to highlight the relative strengths and limitations of each method. Section 7 concludes with an outlook on future developments, including hybrid metrology systems and the challenges posed by next-generation applications.

## 2. Comparison of Representative Technologies

### 2.1. Comparison of Performance Metrics

To provide a consistent framework for evaluating the three principal technologies in this review, their key performance attributes and domain-specific suitability are compared. The comparison draws on both their physical principles and representative implementations reported in the literature.

Table 1 presents a structured comparison of the main performance indicators for the Laser Interferometer (LI), Grating Interferometer (GI), and Time Grating Sensor (TGS) The LI remains the benchmark, achieving picometer-level resolution and accuracies often better than 0.1 nm over ranges exceeding 1 m. Such performance, however, depends on extensive environmental compensation to address refractive index variations and thermal drift, and is typically linked to large system size and high cost. The GI has developed into a robust and practical alternative. Its accuracy is limited by the quality of the grating scale, but it provides clear advantages in compactness, reduced sensitivity to environmental effects, and suitability for multi-DOF measurements with planar gratings. The TGS, based on a space-to-time conversion principle, is still emerging. Its main strengths are miniaturization potential and strong resistance to environmental disturbances. At present, its resolution is at the nanometer level; while higher accuracy is expected, the technology remains at an exploratory stage. These comparisons underscore that precision nanometrology inherently involves trade-offs between ultimate accuracy, environmental robustness, system complexity, and scalability. While previous reviews have typically focused on single technologies in isolation, the present analysis emphasizes their relative strengths and limitations within a unified framework, which is essential for guiding both system design and application-oriented selection.

Building on these characteristics, Table 2 outlines the rationale for selecting each technology in representative applications, showing how requirements shape the choice of sensor. In ultra-precision domains such as semiconductor lithography, the LI remains dominant because of its accuracy and compatibility with vacuum conditions, making it indispensable for stage metrology. In industrial environments such as precision machine tools, the roles are more distinct: the LI is often used for calibration, while the GI has become the preferred solution for integrated real-time feedback due to its robustness and cost–performance balance. The TGS is increasingly viewed as a candidate for these environments, valued for its resistance to vibration and contamination. Where compactness and cost are critical, the LI is less practical. The GI offers a wide set of proven solutions for embedded use, whereas the TGS holds promise for low-cost implementation driven by electronics rather than advanced nanofabrication, combined with a minimal footprint. In practice, sensor selection is not about identifying a universally superior technology but about matching the trade-offs of each method with the requirements of the application. This perspective reinforces the necessity of cross-domain comparison, as the suitability of each method is not absolute but conditional, depending on the interplay of accuracy demands, environmental conditions, integration constraints, and economic factors.

### 2.2. Comparison of Error Sources and Compensation Strategies

Beyond their fundamental principles, the practical viability of LI, GI, and TGS is determined by their dominant error sources and the requisite compensation strategies. (1) LI systems are highly susceptible to environmental perturbations (i.e., air refractive index) and optical path mixing, which causes periodic nonlinearity. These are actively suppressed using environmental compensation units (applying, e.g., the Ciddor equation) and heterodyne (dual-frequency) architectures, respectively. (2) GI systems are inherently more robust to environmental drift but are sensitive to readhead-to-grating geometric misalignments (e.g., yaw, pitch) and grating scale imperfections. These errors are effectively mitigated using differential read-head designs and precise optomechanical alignment. (3) TGS systems, being non-optical, are immune to air turbulence but are dominated by errors from the physical scale (e.g., PCB etching tolerances) and electromagnetic signal crosstalk. Consequently, their accuracy relies heavily on software-based correction maps generated during calibration against a traceable standard (such as an LI).

### 2.3. Comparison of Measurement Bandwidth/Speed

Beyond static accuracy, the dynamic performance (measurement bandwidth) of a sensor is a critical criterion for industrial applicability. The three technologies exhibit distinct dynamic characteristics. Laser Interferometers, processing high-frequency optical beat signals, routinely achieve very high bandwidths at the MHz level, making them the default choice for high-speed scanning stages (e.g., in lithography). Grating Interferometers also demonstrate high bandwidth, typically spanning the kHz to MHz range. Their dynamic response is generally limited not by the optical principle but by the photodetector electronics and the signal processing throughput. Time Grating Sensors currently offer a comparatively lower bandwidth, often in the kHz level. This limitation is primarily due to the computational processing required for its time-based signal demodulation. However, this bandwidth remains sufficient for many industrial target applications, including the motion control of machine tools and robotic arms.

## 3. Fundamental Principles

### 3.1. Laser Interferometer

Laser interferometer works on the principle of comparing optical path lengths, where coherent light beams interfere after traveling along different paths. The classical form is the Michelson interferometer, which uses a beam splitter to divide a laser beam into a reference arm and a measurement arm. When the two beams recombine, the phase difference appears as an intensity change in the interference pattern, which corresponds to displacement.

Modern systems often use heterodyne designs. In these systems, two frequency-shifted beams with orthogonal polarizations generate a stable beat signal. This improves noise resistance and allows direction detection. Homodyne systems are simpler, but they need extra signal processing to remove direction ambiguity. Both types rely on phase demodulation, usually with quadrature detection, to achieve sub-fringe resolution down to the picometer scale. Laser interferometers are also flexible, as they can measure linear, angular, and multi-axis motion, making them widely used in precision metrology. Next, we introduce the principle of the heterodyne laser interferometer.

#### Measurement Principle of Heterodyne Laser Interferometer

**Figure 3:**
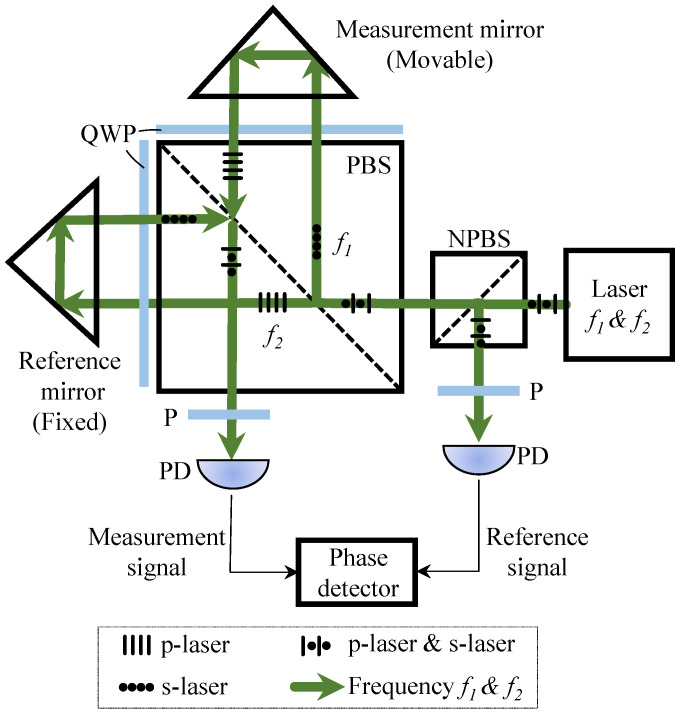
Measurement Principle of Heterodyne Laser Interferometer.

As shown in Figure 3, the system utilizes a laser source emitting two orthogonally polarized frequency components, $f_{1}$ and $f_{2}$. These are separated into a reference beam ($E_{r}$, frequency $f_{1}$) and a measurement beam ($E_{m}$, frequency $f_{2}$). The measurement beam reflects from a moving target, imparting a displacement-dependent phase shift L(t):

$$E_{m}\left(t\right)=A_{m}\cos\left(2\pi f_{2}t-\frac{4\pi nL(t)}{\lambda_{2}}+\phi_{m0}\right)$$

The reference beam is expressed as:

$$E_{r}\left(t\right)=A_{r}\cos\left(2\pi f_{1}t+\phi_{r}\right)$$

The two beams are recombined, producing a heterodyne beat signal on the detector:

$$S_{meas}\left(t\right)\propto \cos\left(\left(2\pi f_{1}t+\phi_{r}\right)-\left(2\pi f_{2}t-\frac{4\pi nL(t)}{\lambda_{2}}+\phi_{m0}\right)\right)$$

The phase is following:

$$\Phi_{meas}\left(t\right)=2\pi \left(f_{1}-f_{2}\right)t+\frac{4\pi nL(t)}{\lambda_{2}}+\phi_{const1}$$

A reference signal is obtained by mixing $f_{1}$ and $f_{2}$, and the phase meter computes the differential phase. The common carrier term $2\pi (f_{1}-f_{2})t$ cancels, suppressing laser frequency noise. Thus the displacement is:

$$L\left(t\right)=\frac{\Delta \Phi (t)}{2\pi }\cdot \frac{\lambda_{2}}{2n}$$

### 3.2. Grating Interferometer

Grating interferometer relies on interference between diffracted beams from a periodic grating illuminated by coherent light. A simple design uses a reflective or transmissive grating to produce several diffracted orders. When these orders overlap, they form an interference signal that depends on the relative displacement between the grating and the detector. The sensitivity is related to the grating pitch.

There are single-diffraction and multi-diffraction systems. More advanced designs, such as Littrow setups or crossed-grating layouts, use multiple diffraction stages or orthogonal paths to improve sensitivity and support multi-degree-of-freedom measurements. A key advantage of grating interferometers is their compact structure and reduced sensitivity to environmental changes. Because they are referenced to the physical grating scale rather than to free-space path length, they are less affected by temperature-driven refractive index variations and are well suited for embedded sensor applications. Next, we introduce the principle of the heterodyne grating interferometer.

#### Measurement Principle of Heterodyne Grating Interferometer

**Figure 4:**
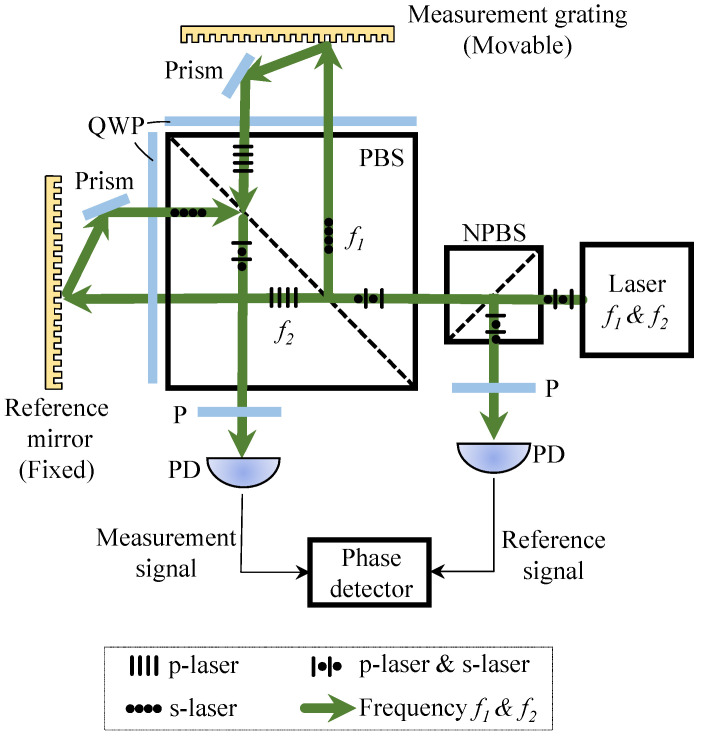
Measurement Principle of Heterodyne Grating Interferometer.

As shown in Figure 4, two beams of frequencies $f_{1}$ and $f_{2}$ impinge on a diffraction grating at ±θ. A displacement x(t) induces opposite phase shifts:

$$\Delta \phi_{1}\left(t\right)=+\frac{2\pi x(t)}{d},\;\;\;\Delta \phi_{2}\left(t\right)=-\frac{2\pi x(t)}{d}$$

where *d* is the grating period. After recombination, the measurement phase is:

$$\Phi_{meas}\left(t\right)=2\pi \left(f_{1}-f_{2}\right)t+\frac{4\pi x(t)}{d}+\phi_{const1}$$

As in the laser case, subtracting the reference phase cancels the carrier frequency and isolates the displacement information. The result is:

$$x\left(t\right)=\frac{\Delta \Phi (t)}{2\pi }\cdot \frac{d}{2}$$

where d/2 corresponding to one 2π phase cycle.

### 3.3. Time Grating Sensor

Time grating sensors differ from the two optical methods because they encode displacement information into time-domain electrical signals. Instead of using optical interference, TGS applies alternating orthogonal electric fields to encode position as a phase shift in time waveforms. These signals are picked up through capacitive electrodes, and displacement is calculated from the time difference between waveform peaks or zero crossings.

This space-to-time conversion allows high-resolution measurement over long ranges without strict optical alignment or fine-pitch gratings. Unlike optical encoders, the performance of TGS does not depend on advanced nanofabrication, which makes them easier to scale at low cost. Their compact size, resistance to ambient light, and ability to support planar or cylindrical layouts make TGS a promising option for both linear and angular displacement sensing. Next, we introduce the principle of the time grating sensor.

#### Measurement Principle of the Time Grating Sensor

**Figure 5:**
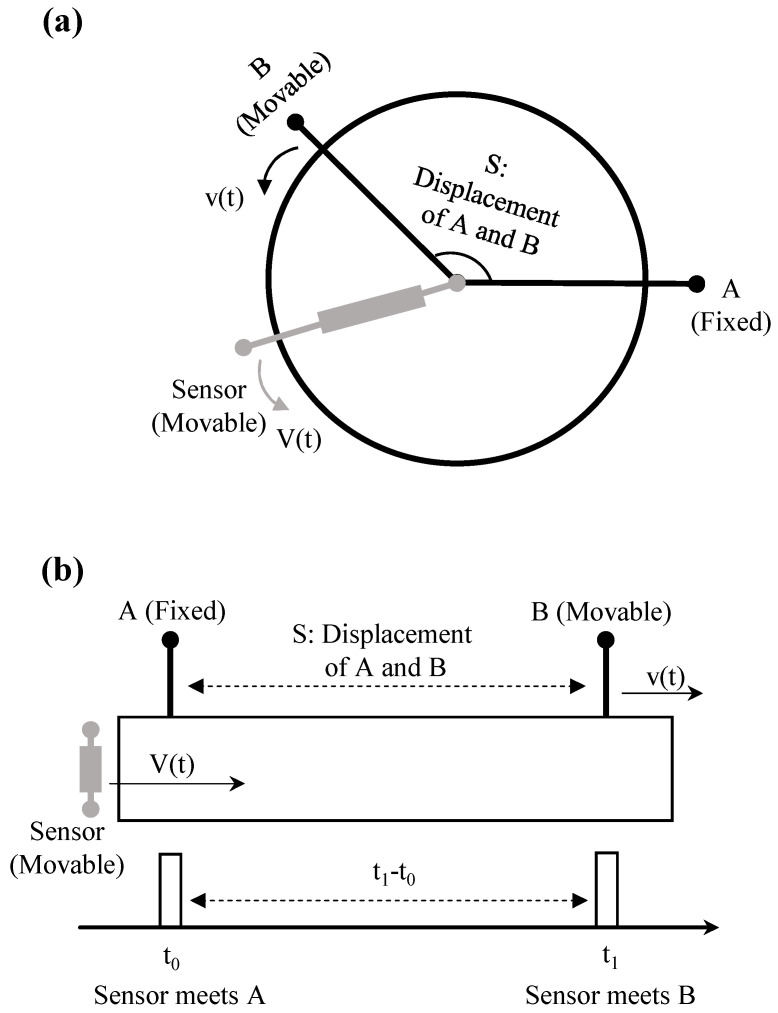
Measurement Principle of the Time Grating Sensor. (**a**) Schematic diagram of time grating sensor based on rotation principle. (**b**) Schematic diagram of time grating sensor based on translational principle.

As shown in Figure 5, the principle is analogous to an analog clock: a scanner with constant angular velocity Ω converts spatial displacement into a measurable time interval ΔT. During a sweep, the time interval between passing a fixed reference marker and a moving measurement marker is:

x(t)=Ω·ΔT(t)

In practice, ΔT is quantified by counting *N* cycles of a stable clock with period $T_{clk}$, giving:

$$x\left(t\right)=\Omega \cdot N\cdot T_{clk}$$

Thus, the time grating is formed by the clock pulses, and the resolution depends on clock stability and scanner velocity rather than a physical grating dimension.

### 3.4. Comparison of Fundamental Mechanisms

At the most basic level, all three technologies convert mechanical displacement into phase changes that can be measured. The way this conversion is achieved, however, differs greatly.

Laser interferometers rely on differences in free-space optical path length, with phase information extracted from interference fringes of coherent light. Grating interferometers use periodic gratings to diffract light, and the interference signal depends on the relative motion of the grating. Time grating sensors encode position as phase shifts in time-domain electrical signals produced by orthogonal electric field modulation.

Laser interferometers provide the highest resolution and direct traceability but are sensitive to the environment and often require large systems. Grating interferometers reduce alignment sensitivity and allow more compact designs, but they depend on the precision of grating fabrication. Time grating sensors reach good performance mainly through electrical design and signal processing, giving them additional flexibility in system design.

In the following sections, we review representative advances and applications of each technology, with attention to structural configurations, signal processing strategies, and system-level performance.

## 4. Advances in High-Precision Laser Interferometer

**Figure 6:**
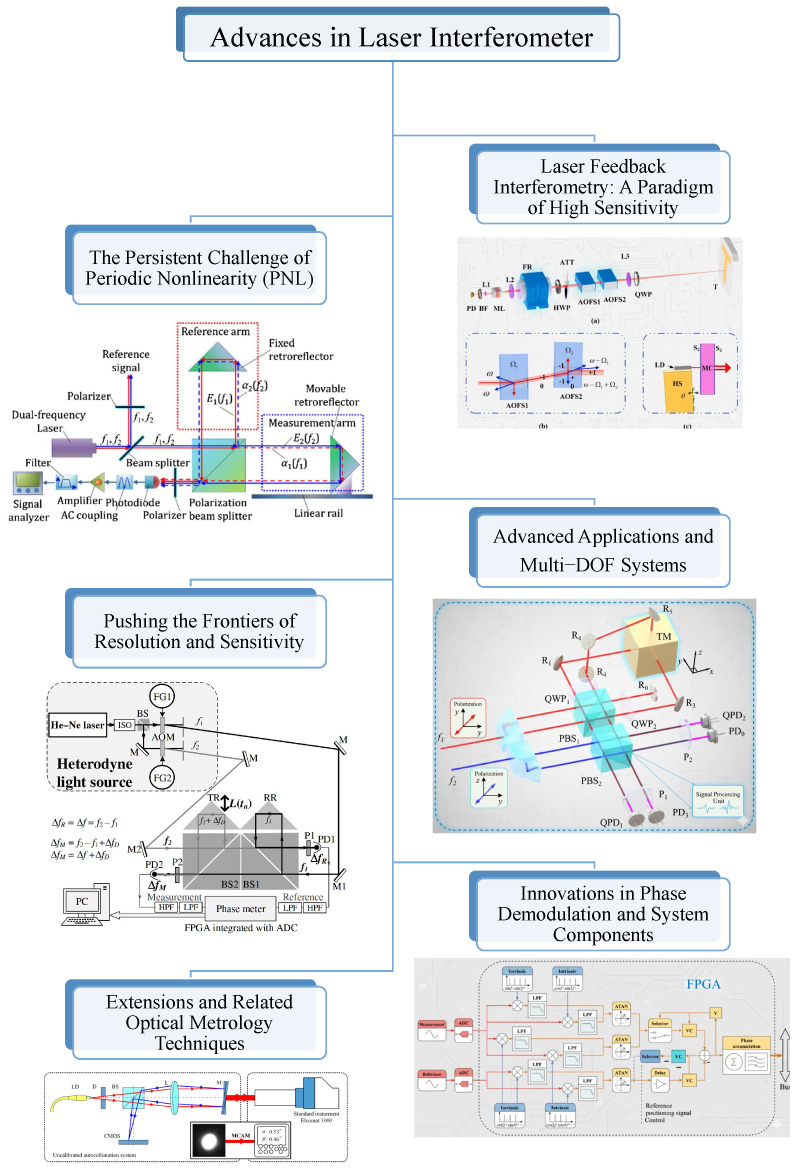
Advances in Laser Interferometer. The Persistent Challenge of Periodic Nonlinearity (PNL), copyright Optica, reproduced with permission from [65]. Pushing the Frontiers of Resolution and Sensitivity, copyright Optica, reproduced with permission from [48]. Innovations in Phase Demodulation and System Components, copyright IEEE, reproduced with permission from [66]. Laser Feedback Interferometor: A method of High Sensitivity, copyright Elsevier, reproduced with permission from [50]. Advanced Applications and Multi-DOF Systems, copyright IEEE, reproduced with permission from [67]. Extensions and Related Optical Metrology Techniques, copyright Optica, reproduced with permission from [68]. This figure visually summarizes key research thrusts in advanced laser interferometry, compiling representative schematics for dominant challenges (PNL), sensitivity enhancement (laser feedback), and system integration (multi-DOF).

Laser interferometer is a core technology in modern dimensional metrology, enabling displacement measurements with resolutions that have advanced from the nanometer to the picometer scale. It is widely applied in semiconductor lithography, precision manufacturing, and fundamental physics. As shown in Figure 6, this section reviews the main developments in the field, from early system-level improvements to the control of picometer-level error sources and the introduction of new interferometric methods.

### 4.1. Foundational Advances and System-Level Integration

The pursuit of nanometer-scale accuracy in industrial applications has driven much of the early and ongoing innovation. A comprehensive review by Bobroff N. (1993-IBM-USA, [3]) articulated the state-of-the-art, emphasizing the critical interplay between optical configuration, environmental stability, and electronic signal processing to achieve accuracies on the order of $10^{-7}$ over dynamic ranges exceeding $10^{6}$. This work laid out the primary challenges, including air refractive index fluctuations and the need for robust phase measurement, that would occupy researchers for decades.

Translating this potential into a robust industrial tool was demonstrated by Demarest F. C. (1998-Zygo-USA, [69]), who detailed a heterodyne interferometer electronic system for semiconductor manufacturing. This system achieved an impressive 0.31 nm resolution at speeds up to 2.1 m/s, enabled by a 20 MHz heterodyne frequency, fiber optic beam delivery, and custom ASIC-based phase meters, showcasing a complete, high-performance solution for demanding industrial environments.

### 4.2. The Persistent Challenge of Periodic Nonlinearity (PNL)

Perhaps the most significant and persistent limitation to achieving sub-nanometer and picometer accuracy in heterodyne interferometor is the periodic nonlinearity (PNL), also known as cyclic error. This error arises primarily from frequency and polarization mixing due to imperfect optical components. Research in this area can be broadly categorized into modeling, compensation, and suppression through novel optical design.

#### 4.2.1. Analysis, Modeling, and Component-Level Effects

Understanding the root causes of PNL is a prerequisite for its mitigation. Keem T. et al. (2004-Korea, [70]) employed Jones matrix analysis to model the influence of polarizing beam splitters (PBS) and waveplates on PNL, demonstrating that non-ideal optical characteristics are a primary contributor. Similarly, a detailed analysis by Yan L. et al. (2015-Zhejiang University of Technology-China, [71]) established a direct functional relationship between the misalignment of a thin-film PBS and the resulting PNL, identifying yaw error as the most critical factor.

More recently, research has uncovered more subtle error mechanisms. Work from the team of Tan J. (2015-Harbin Institute of Technology-Hu P., [72]) proposed a new model showing that even without direct frequency mixing between the two primary laser beams, multi-order Doppler frequency shifts from the measurement beam itself can cause higher-order PNL. This was further explored by identifying ghost reflections from components like corner-cube retroreflectors as a source of complex periodic errors (Hu P. et al., 2017-Harbin Institute of Technology-Tan J., [73]). Later work confirmed that the coupling of ghost reflections and frequency mixing severely degrades the efficacy of standard correction algorithms and is a dominant source of residual PNL at the picometer level (Fu H. et al., 2018-Harbin Institute of Technology-Tan J., [74]). Further complicating matters, intermodulation between signals was identified as a source of anomalous harmonics in the frequency domain, which are not explained by traditional models and point to nonlinearities within the signal detection and processing chain itself (Fu H. et al., 2017-Harbin Institute of Technology-Tan J., [65]).

#### 4.2.2. Error Compensation and Real-Time Correction

Given the presence of PNL, numerous compensation techniques have been developed. An early and widely adopted method presented by Eom T. B. et al. (2002-Korea, [75]) used an elliptic fitting algorithm on the quadrature Lissajous figures to correct for phase errors, successfully reducing a 3.8 nm error down to 1.0 nm. A similar approach was validated by Schmitz T. L. et al. (2006-University of Florida-USA, [76]), who demonstrated that a first-order digital correction algorithm could reduce PNL from 5.4 nm to 0.1 nm under both constant and non-constant velocity conditions.

The demand for real-time correction led to FPGA-based solutions. Kim J. A. et al. (2009-Korea, [77]) developed an FPGA module that implemented a real-time correction based on simple arithmetic operations, achieving results comparable to elliptic fitting with a cycle time of just 4.4 µs. The group of Tan J. (2015-Harbin Institute of Technology-Hu P., [78]) further advanced this by developing an FPGA-based method using simple peak detectors to estimate signal parameters and correct for variable periodic errors in real-time, reducing errors to below 0.6 nm even at high speeds. This work was later enhanced with a dual-channel orthogonal demodulation technique implemented on an FPGA, which achieved a measurement accuracy better than 2 pm by using an internal reference signal, making it independent of target motion (Fu H. et al., 2018-Harbin Institute of Technology-Tan J., [79]).

Passive compensation methods have also been explored. Ahn J. et al. (2009-Korea, [80]) demonstrated a technique to reduce PNL from 3.75 nm to 0.36 nm simply by re-adjusting the axial angles of waveplates based on the measured characteristics of the PBS, avoiding complex data processing.

#### 4.2.3. Novel Optical Configurations for PNL Suppression

The most elegant solution to PNL is to prevent it optically. Joo K. N. et al. (2009-Netherlands, [81]) introduced a novel design that spatially separated the two frequency beams, which effectively prevented polarization mixing and reduced PNL to less than 0.15 nm. They later refined this into a simple, industry-ready configuration with a demonstrated noise floor of 20 pm and no detectable PNL (Joo K. N. et al., 2010-Netherlands). The team at PTB (2012-Germany-Weichert C., [82]) developed a similar plane-parallel plate interferometer with spatially separated beams, achieving a PNL of less than ±10 pm when cross-calibrated against an X-ray interferometer.

The group of Tan J. (2016-Harbin Institute of Technology-Cui J., [83]) identified quarter-waveplate (QWP) polarization crosstalk as a primary error source and designed an interferometer using a non-polarizing beam splitter and a balanced interference of two circularly polarized beams to minimize this effect, achieving a corrected PNL of 0.2 nm. A later design featuring a fully balanced optical path with spatially separated beams demonstrated exceptional thermal stability (1.2 nm/°C) and no detectable PNL at the 13 pm level, making it suitable for long-term, high-stability applications (Fu H. et al., 2018-Harbin Institute of Technology-Tan J., [84]). More recently, Meskers A. J. H. et al. (2014-Netherlands, [85]) designed two novel interferometers that are inherently insensitive to the input polarization state of the heterodyne source, a significant practical advantage.

### 4.3. Pushing the Frontiers of Resolution and Sensitivity

Beyond mitigating systematic errors, a major research thrust has been to enhance intrinsic resolution and sensitivity into the picometer (pm) and even femtometer (fm) domains.

Multi-pass interferometor is a direct way to amplify displacement. Pisani M. (2008-Italy, [43]) developed a multi-reflection Michelson interferometer that achieved a 60× optical path multiplication, yielding a resolution of approximately 4 pm. A subsequent homodyne version achieved over 100× amplification with a noise spectral density below 20 fm, ideal for vibration analysis (Pisani M., 2009-Italy, [86]).

Achieving such resolutions requires exceptionally sensitive phase meters. Hsu M. T. L. et al. (2010-Australia, [87]) developed a fully digital RF phase meter based on a phase-locked loop (PLL) architecture, which, when combined with an active Sagnac interferometer, demonstrated 0.5 pm displacement sensitivity above 1 Hz. Another approach by Park Y. et al. (2011-Korea, [44]) used an AOM in a double-pass configuration to measure small-amplitude vibrations with a sensitivity approaching the quantum noise limit, achieving a minimum displacement measurement of 0.5 pm. A system capable of absolute vibration amplitude measurement with a noise floor of 7.1 fm at 21 MHz was demonstrated by Leirset E. et al. (2013-Norway, [88]).

Further progress was made by Dong Nguyen T. et al. (2020-Japan, [48]), who combined a single-path heterodyne interferometer with an FPGA-based PLL phase meter to achieve 11 pm measurement accuracy in a vacuum. Most recently, a design for high-speed, high-resolution interferometor featuring a bias-locked dual-frequency laser source was proposed, targeting picometer resolution at several meters per second (Yang H. et al., 2020-Harbin Institute of Technology-Tan J., [89]).

### 4.4. Innovations in Phase Demodulation and System Components

The method of extracting phase from the beat signal is critical for both speed and accuracy. To overcome the computational load of traditional arctangent calculations, Choi H. et al. (2005-Korea, [90]) converted the interference signal into a PWM (Pulse Width Modulation) signal, enabling rapid displacement calculation using analog circuits and a DSP. To enable full-field, real-time measurements, Kimachi A. et al. (2007-Japan, [91]) utilized a three-phase correlation image sensor (3PCIS) to simultaneously demodulate the amplitude and phase images of an interferogram at standard video frame rates. The group of Tan J. (2023-Harbin Institute of Technology-Hu P., [66]) developed a digital dual-frequency comb method for phase measurement, improving accuracy by over 50% during high-speed motion compared to PLL methods and achieving a phase measurement accuracy equivalent to 19 pm.

Fiber optic coupling offers flexibility but introduces errors. Ellis J. D. et al. (2011-Netherlands, [92]) designed a fiber-coupled interferometer that uses spatially separated input beams and a reference signal tapped before the fiber to eliminate fiber-induced drift and PNL. This concept was further validated by Meskers A. J. H. et al. (2014-Netherlands, [93]), proving that sub-nanometer uncertainty is achievable with fiber-delivered source frequencies.

Innovations in laser sources are also critical. The team of Li J. et al. (2019-Tsinghua University-Tan Y., [94]) developed a dual-frequency solid-state microchip laser and implemented a PID closed-loop control system to continuously tune and stabilize the frequency difference, making it a viable compact source for heterodyne interferometor.

### 4.5. Laser Feedback Interferometor: A Method of High Sensitivity

A distinct and highly sensitive method is laser feedback interferometor (LFI), or self-mixing interferometor, where light scattered from a target re-enters the laser cavity, modulating the laser’s output. The research group of Tan Y. at Tsinghua University has been at the forefront of this technology. They developed a self-mixing technique using orthogonally polarized beams for the measurement and reference paths, achieving <2 nm resolution with excellent environmental robustness (Zhang S. et al., 2016, [95]). This was extended to simultaneous, non-contact measurement of in-plane and out-of-plane displacement of a non-cooperative target (Zhu K. et al., 2017, [47]).

The extreme sensitivity of LFI was highlighted by demonstrating its use as a remote eavesdropping system, capable of reconstructing speech by detecting vibrations of objects from 200 m away (Xu Z. et al., 2021, [96]). The fundamental coherent detection limit of this technique was shown to reach an incredible 0.5 photons/second, six orders of magnitude more sensitive than traditional heterodyne detection (Tian M. et al., 2022, [97]). They have also applied LFI to develop novel measurement systems, including an all-path compensated system to eliminate dead-path error for long-range (10 m) measurement (Xu L. et al., 2018, [98]), a dual-beam differential method for non-contact linear and angular displacement measurement (Xu X. et al., 2023, [99]), a frequency-scanning LFI for long-distance (152 m) ranging (Wang Y. et al., 2022, [100]), and a fully fiberized LFI for high-sensitivity measurements over a 300 m distance (Wang Y. et al., 2021, [101]). Most recently, an anti-jamming LFI was designed that uses the laser cavity as a high-rejection filter to operate reliably even under high-power laser interference (Zhou B. et al., 2024, [50]). A comprehensive review of frequency-shifted optical feedback techniques was also provided by the group (Zhu K. et al., 2018, [102]).

### 4.6. Advanced Applications and Multi-Degree-of-Freedom (Multi-DOF) Systems

The maturity of laser interferometor is reflected in its application to cutting-edge scientific instruments and complex multi-DOF systems. For the Joule balance experiment, a six-axis heterodyne interferometer system was developed to measure the relative displacement of coils with 0.31 nm uncertainty (Bai Y. et al., 2017-Harbin Institute of Technology-Tan J., [103]). For space-based gravitational wave detection, a test mass motion readout system capable of 6-DOF measurement was developed using a phase-locked dual-frequency source and differential wavefront sensing (Xu X. et al., 2024-Tsinghua University-Tan Y., [67]). Another method for high-precision 3-DOF angle measurement was achieved using a transmission grating and combined mirrors (Ren W. et al., 2022-Harbin Institute of Technology-Tan J., [104]). Yang F. et al. (2019-Tsinghua University-Wang L., [105]) expanded the angular measurement range of a 3-DOF system by replacing a quadrant detector with a fiber bundle. The unique challenge of measuring the parameters of the laser source and optics themselves was addressed by Lu Z. et al. (2018-Harbin Institute of Technology-Tan J., [106]) using a direct beat frequency measurement method. Similarly, Wang J. et al. (2022-Harbin Institute of Technology-Tan J., [107]) developed a method for the equivalent measurement and real-time compensation of errors caused by intensity changes, reducing a 220 pm error to less than 40 pm.

### 4.7. Extensions and Related Optical Metrology Techniques

The principles of interferometor and precision optics have been applied to a wide range of measurement problems beyond simple displacement. This includes a system for measuring optical sound fields (Ishikawa K. et al., 2020-Japan, [108]), a contact-type displacement sensor based on FMCW laser interferometor (Sun B. et al., 2021, [109]), and a Michelson-based system for detecting defects in industrial injection-molded products (Xu X. et al., 2024-Zhejiang University, [110]). In angular measurement, high-resolution measurement over a large 26° range was achieved using a birefringent heterodyne interferometer (Hsieh H. L. et al., 2016-Taiwan, [46]). The accuracy of autocollimators, another key tool for angle metrology, has been improved by modeling the effect of lens aberrations (Shi J. et al., 2023-Harbin Institute of Technology-Tan J., [111]) and by using multi-scale convolutional neural networks (MSCNN) to correct for nonlinear errors (Shi J. et al., 2022-Harbin Institute of Technology-Tan J., [68]). This list is by no means exhaustive but highlights the breadth of innovation spurred by the core technologies reviewed herein.

### 4.8. Summary and Future Prospects

Laser interferometer has evolved from single-axis reference setups to integrated, environmentally compensated, and multi-dimensional platforms [112,113,114,115,116,117,118]. With advances in optical design, signal processing, and traceability, modern systems now support both fundamental metrology and industrial process monitoring [119,120,121,122,123,124,125].

The move toward multi-DOF, long-range, and absolute systems reflects the rising demands of applications such as semiconductor lithography and spaceborne instruments. Current research directions include: 1. Modular optical heads for compact and flexible installation; 2. SI-traceable, self-calibrating designs based on frequency combs and optical synthesizers; 3. Real-time digital processing using FPGA and machine learning for error reduction; 4. Hybrid platforms that combine interferometers with encoders, gratings, or capacitive sensors.

By combining these approaches, future interferometers are expected to provide greater robustness, scalability, and adaptability, reinforcing their role as a central component of precision metrology in advanced industrial and scientific applications.

## 5. Advances in Grating Interferometer for Precision Motion Metrology

**Figure 7:**
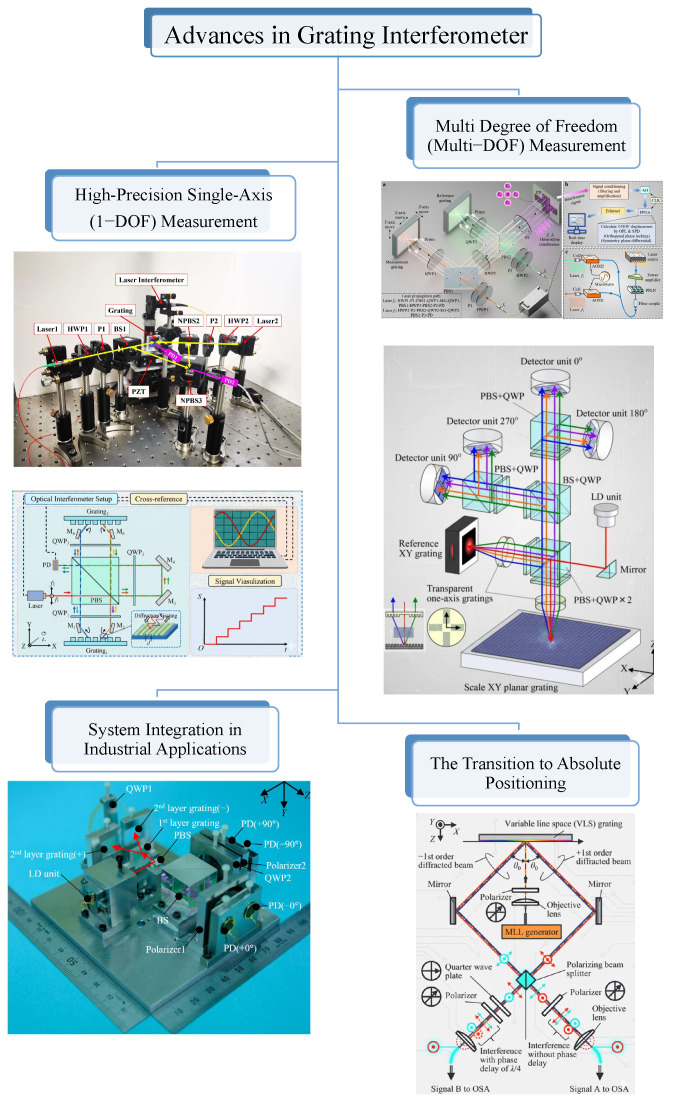
Advances in Grating Interferometer. High-Precision Single-Axis (1-DOF) Measurement, copyright IEEE/Wiley, reproduced with permission from [56,57]. Multi-Degree-of-Freedom (Multi-DOF) Measurement, copyright Elsevier, reproduced with permission from [41,126]. System Integration in Industrial Applications, copyright Optica, reproduced with permission from [127]. The Transition to Absolute Positioning, copyright IOP, reproduced with permission from [128]. This figure compiles representative systems that illustrate the primary development trends in grating interferometry, progressing from high-precision 1-DOF setups to integrated multi-DOF and absolute measurement solutions.

Grating interferometers, often referred to as optical encoders, are now widely used in precision displacement measurement, with applications ranging from machine tools to semiconductor lithography stages. Their main advantages compared with laser interferometers are compact size, lower sensitivity to environmental changes, and natural suitability for multi-axis measurement. These benefits have driven continuous research and development. As shown in Figure 7, this section reviews the key progress, from early single-axis designs to advanced multi-degree-of-freedom (Multi-DOF) and absolute positioning systems.

### 5.1. High-Precision Single-Axis (1-DOF) Grating Interferometor

The foundation of grating-based metrology lies in achieving nanometer and sub-nanometer resolution for linear displacement.

#### 5.1.1. Foundational Concepts and System-Level Performance

The advantages of grating interferometor were articulated early on by Teimel (1992-Heidenhain-Germany, [129]), who introduced a system demonstrating 1 nm resolution over a 200 mm range, highlighting its superior environmental stability compared to open-path laser interferometers. This work established grating encoders as a robust solution for industrial precision machinery. The conceptual framework for pushing these systems to their ultimate limits was later explored by Heilmann R. K. (2004-MIT-USA, [51]), who proposed using advanced nanorulers—gratings fabricated with 1 nm placement repeatability over 300 mm—to create encoders with sub-nanometer accuracy, addressing the metrological needs of the burgeoning nanotechnology sector.

#### 5.1.2. Pushing for Sub-Nanometer Accuracy and PNL Suppression

A primary focus of 1-DOF research has been to enhance resolution and suppress PNL. A heterodyne grating interferometer using a phase-locked amplifier achieved a practical resolution of 0.2 nm, demonstrating the potential for sub-nanometer performance (Lee J.-Y., 2007-Taiwan, [130]). Another quasi-common-path heterodyne design achieved <14 nm stability over one hour and <1 nm repeatability (Hsieh H.-D., 2010-Taiwan, [131]). Using a Littrow configuration to increase measurement range, a heterodyne common-path grating interferometer demonstrated an estimated resolution of 0.15 nm and stability two orders of magnitude better than a commercial laser interferometer in a standard laboratory environment (Wu C.-C., 2013-Taiwan, [132]).

A critical technique for suppressing PNL, mirroring developments in laser interferometor, is the spatial separation of frequency beams. The German metrology institute PTB (2017-Germany-Guan J., [53]) developed a differential heterodyne encoder with spatially separated input beams that achieved PNL of less than 30 pm without correction and a static stability of 100 pm over one hour. A similar space-separated heterodyne configuration was developed by the group of Tan J. (2017-Harbin Institute of Technology-Xing X., [54]), which reduced PNL to below 0.086 nm. More recently, a wavelength-stabilized, quasi-common-path heterodyne system employing a special grating structure reduced PNL to below ±0.3 nm, achieving 0.4 nm repeatability and ±30 nm long-term stability over 8 h (Wang G. et al., 2024-Tsinghua University and NUDT, [56]).

#### 5.1.3. Novel Configurations for Enhanced Performance

Innovations in optical configuration have further improved performance. A symmetric heterodyne design was proposed to enhance signal contrast and signal-to-noise ratio, achieving a theoretical resolution of 12 pm (Lin C. et al., 2015-NUDT-China, [133]). For applications in scanning interference lithography, a compact (48 × 48 × 18 mm) heterodyne Littrow interferometer was designed with a dead-path error of only 7.59 nm, showcasing high environmental robustness (Wang L. et al., 2017-Tsinghua University-Zhu Y., [134]). To improve tolerance to angular disturbances during linear motion, a symmetric Littrow structure with two gratings was proposed, which used common-mode rejection to increase angular tolerance (Zhou W., 2025-CIOMP-China, [57]). A dual-grating Littrow configuration was also developed to achieve a four-fold optical subdivision, reducing measurement error by 30% and improving resolution by 50% compared to traditional designs (Zhou W., 2024-CIOMP-China, [135]).

### 5.2. Multi-Degree-of-Freedom (Multi-DOF) Measurement

A key advantage of grating interferometor is its ability to measure multiple DOFs with a single readhead and grating, a critical need for planar stages, robotics, and complex machinery.

#### 5.2.1. Two-Degree-of-Freedom (2-DOF) Systems

Initial 2-DOF work focused on measuring two orthogonal in-plane axes (X-Y). A heterodyne system was demonstrated with 0.5 nm resolution and sub-picometer sensitivity for 2D positioning (Hsu C.-C., 2008-Taiwan, [136]). This was followed by a quasi-common-path design capable of 2D in-plane measurement with resolutions down to 4.5 pm (Hsieh H.-D., 2011-Taiwan, [137]).

The simultaneous measurement of in-plane (x) and out-of-plane (z) motion is a particularly important configuration. Early work demonstrated this capability for measuring the position and straightness of a linear stage (Kimura A. et al., 2010-Tohoku University and Tsinghua University, [138]). Further research explored a dual-diffraction configuration insensitive to grating tilt (Feng C. et al., 2013-Tsinghua University-Zeng L., [139]), and a heterodyne system capable of 1.63 nm (in-plane) and 0.75 nm (out-of-plane) resolution for lithographic applications (Wang L. et al., 2014-Tsinghua University-Zhu Y., [140]). A dual-diffraction grating system was also developed for independent X and Z measurement (Lu Z. et al., 2016-Harbin Institute of Technology-Tan J., [141]). A simple and compact Littrow configuration achieved 0.27 nm and 0.18 nm theoretical resolution in the x and z directions, respectively (Lv Q., 2018-CIOMP-China, [142]). To enhance stability, a fiber-coupled heterodyne system with a reference beam path was designed, achieving 0.246 nm and 0.465 nm stability over 30 s for x and z axes (Yang F. et al., 2019-Tsinghua University-Zhu Y., [143]). Recently, a space-separated heterodyne design demonstrated 2.5 nm stability and the ability to distinguish 5 nm steps in both x and z directions (Chang D. et al., 2022-Harbin Institute of Technology-Tan J., [144]). The Fizeau configuration was used to extend the Z-axis measurement range to ±1.5 mm, far beyond traditional surface encoders (Hong Y. et al., 2022-Tohoku University, [145]), a concept that was later improved to reduce crosstalk error (Hong Y. et al., 2022-Tohoku University, [146]) and then extended to a 13 mm Z-range using a simple Littrow configuration (Hong Y. et al., 2024-Tohoku University, [147]).

#### 5.2.2. Three-Degree-of-Freedom (3-DOF) Systems

The first 3-DOF grating interferometer, capable of measuring X, Y, and Z displacement simultaneously using a 2D XY grid, was proposed by Gao W. (2007-Tohoku University-Japan, [148]). This concept was refined to achieve sub-nanometer resolution in all three axes, with interpolation errors less than 1% of a signal period (Kimura A., 2012-Tohoku University-Japan, [126]). Other approaches include a heterodyne system using a transmissive 2D grating and electro-optic modulation (Hsieh H.-D., 2013-Taiwan, [149]), and a compact system using a single 1D grating and multiple diffracted beams to measure X, Y, and Z motion (Lin J. et al., 2017-Harbin Institute of Technology-Tan J., [150]). A compact design based on a pyramidal prism was developed to address space constraints in multi-DOF systems (Wang S. et al., 2023-Tsinghua University, [151]). A recent non-Michelson type 3-axis system was proposed using four linear gratings to overcome the size limitations of 2D planar gratings (Sato R., 2025-Tohoku University-Japan, [152]). An alternative approach with a rotationally symmetric optical configuration demonstrated a large Z-axis stroke of 7.6 mm and a high rotational tolerance of 0.75 degrees (Chen X. et al., 2025-Shanghai Jiao Tong University-China, [153]). For the most demanding applications, a zero-dead-path, 3-DOF heterodyne system was developed with a crosstalk compensation algorithm, targeting next-generation lithography and atomic-scale manufacturing (Cui C. et al., 2025-Tsinghua University, [41]).

#### 5.2.3. Multi-Axis Angular and 5/6-DOF Systems

Extending the technology to full 6-DOF measurement (X, Y, Z, pitch, yaw, roll) represents the pinnacle of motion metrology. An early approach to 3-DOF *angle* measurement was developed by replacing the mirror in an autocollimator with a diffraction grating (Saito Y., 2009-Tohoku University-Japan, [154]), a design later improved to achieve 0.01 arc-second resolution (Gao W., 2011-Tohoku University-Japan, [155]).

The first integrated 6-DOF measurement systems were significant breakthroughs. Lee C. B. et al. (2011-Korea, [156]) developed a single-unit optical encoder capable of measuring all 6-DOF motion errors of a linear stage, achieving <0.4 nm displacement and <0.03 arc-second angular resolution. The same group later performed a detailed uncertainty analysis of the various error sources in their 6-DOF system (Lee C. B., 2012-Korea, [157]). The group at Tohoku University (Li X. et al., 2013, [52]) proposed the first 6-DOF surface encoder using a single-probe configuration. Then, Li X. (2014, [158]) developed a 6-DOF surface encoder using a three-probe configuration, later investigating methods to compensate for the significant crosstalk errors between axes (Li X., 2014, [159]). This was further improved by optimizing the optical design to reduce polarization-leakage-induced crosstalk by over two orders of magnitude (Matsukuma H., 2019-Tohoku University-Japan, [160]). A five-dimensional system measuring 2D displacement and 3D angles was also developed at CIOMP (Lv Q., 2020-China, [161]). For positioning in synthetic aperture optics, a dual-channel 6-DOF encoder was developed where angle and displacement modules share a common optical path (Yu K. et al., 2021-Tsinghua University, [162]). A new posture representation method, the “fused-like angle,” was proposed to overcome the sequence-dependency and errors of traditional Euler angles in 6-DOF systems (Chang D. et al., 2021-Harbin Institute of Technology-Tan J., [163]).

### 5.3. The Transition to Absolute Positioning

While incremental encoders are highly precise, many applications require absolute position information. This has led to the development of hybrid and novel absolute encoders. A common approach is the dual-probe hybrid method, where one probe reads a coarse absolute track (often using a mask) and a second probe reads the fine incremental track. This was used to achieve 10 nm precision absolute positioning (Shi Y. et al., 2019-Tsinghua University, [164]) and later refined for commercial applications (Shi Y. et al., 2020-Tsinghua University, [165]). A similar dual-probe encoder demonstrated 500nm absolute accuracy (Li X., 2016-Tsinghua University, [166]). A 4-DOF absolute system was developed to measure Z-position and three attitude angles simultaneously (Li X. and Wang S. et al., 2022-Tsinghua University, [167]). A more novel approach utilizes a variable line spacing (VLS) grating and a broadband femtosecond laser, where the absolute position is determined from the diffraction angle of the broadband spectrum, a method pioneered by Sato R. et al. (2024-Tohoku University-Japan, [128]).

### 5.4. System Integration in Industrial Applications

The industrial success of grating interferometor is best exemplified by its adoption in semiconductor lithography. ASML developed an innovative stage positioning system for its immersion lithography tools based on 2D grating encoders, enabling sub-nanometer positioning accuracy at very high stage accelerations (de Jong F., 2009-ASML-Netherlands, [168]). A detailed follow-up on their next-generation NXT:1950i platform highlighted how this grating-based metrology system was key to achieving a 2.5 nm overlay error at a throughput of 175 wafers per hour, pushing optical lithography to the 32/22 nm nodes (Castenmiller T., 2010-ASML-Netherlands, [169]). The development of highly integrated [127], multi-DOF measurement systems is a direct response to these industrial demands. For example, a method for real-time 6-DOF displacement calculation and offline geometric calibration was developed specifically for lithography wafer stages, achieving picometer-level calculation accuracy with a latency of only 1.7 µs (Ye W. et al., 2019-Tsinghua University-Zhu Y., [170]). These examples underscore the critical role of advanced grating interferometor in enabling the manufacturing of modern electronics.

The development of nano-grating standard artifacts has attracted increasing attention, as they provide the essential calibration basis for high-precision measurements. Standardized gratings fabricated with ultra-high uniformity are used to validate displacement scales and to evaluate the nonlinearity of interferometric encoders. For example, recent studies have reported one-dimensional and two-dimensional nano-grating artifacts with sub-nanometer line edge roughness and pitch uncertainties on the order of 0.1 nm, enabling traceable calibration of grating-based encoders. The integration of such nano-grating standards with interferometric systems enhances accuracy, reduces systematic errors, and strengthens the reliability of measurement results.

### 5.5. Summary of Grating Interferometor

The technology has developed from being an alternative to laser interferometers into a leading solution for precision motion control [171]. Early work focused on improving single-axis accuracy, followed by the extension to multi-DOF and absolute measurement. Important steps included reducing periodic errors through optical designs such as spatial separation, combining multiple axes into compact readheads, and proposing new absolute encoding methods. Future studies are expected to address error reduction, higher measurement bandwidth [172], intelligent compensation using machine learning, and the use of optical frequency combs for enhanced performance [138,173,174,175,176,177,178]. In addition, the high-performance fabrication of gratings is crucial for measurement performance, and there are currently some representative works [179,180,181,182,183,184,185,186].

From the early work of Teimel in the 1990s to more recent zero-dead-zone, 6-DOF, and absolute systems, the field shows several phases of progress. The first phase confirmed that grating encoders could compete with laser interferometers, especially in terms of robustness and easier alignment. The second phase saw rapid improvements using heterodyne detection, Littrow arrangements, and spatially separated designs, which improved resolution, linearity, and long-term stability [121,143,187,188,189,190,191,192,193,194,195,196,197,198,199]. A third phase has been driven by the needs of semiconductor manufacturing, aerospace, and nanofabrication, leading to multi-DOF grating interferometers. These systems allow simultaneous measurement of in-plane and out-of-plane displacement, as well as integrated angle sensing and cross-talk compensation. More recently, the combination of grating interferometers with absolute encoding has improved reliability, eliminated initialization requirements, and provided direct referencing [9,157,200,201,202,203,204,205,206,207,208,209,210,211,212,213,214,215].

Future directions include [127,216,217,218,219,220,221,222,223,224,225,226,227,228,229,230,231,232]: 1. Higher-dimensional integration: expanding 3-DOF and 6-DOF systems into scalable multi-axis networks for machine tools, wafer stages, and robotics; 2. Monolithic optical integration: combining diffractive and refractive elements into thermally stable substrates to reduce drift and system size; 3. Real-time signal processing: applying FPGA and AI-based algorithms for fast phase extraction and error correction; 4. Hybrid systems: merging grating interferometers with capacitive, inductive, or frequency-comb encoders to combine wide range with high resolution; 5. Application-driven customization: tailoring grating geometries and detection layouts for lithography, precision assembly, or quantum devices.

In summary, grating interferometers have become a central tool in precision metrology. Their flexibility, high resolution, and integration potential ensure an important role in the next generation of stable and scalable measurement systems for both industry and science.

## 6. Advances in Time Grating Sensors

**Figure 8:**
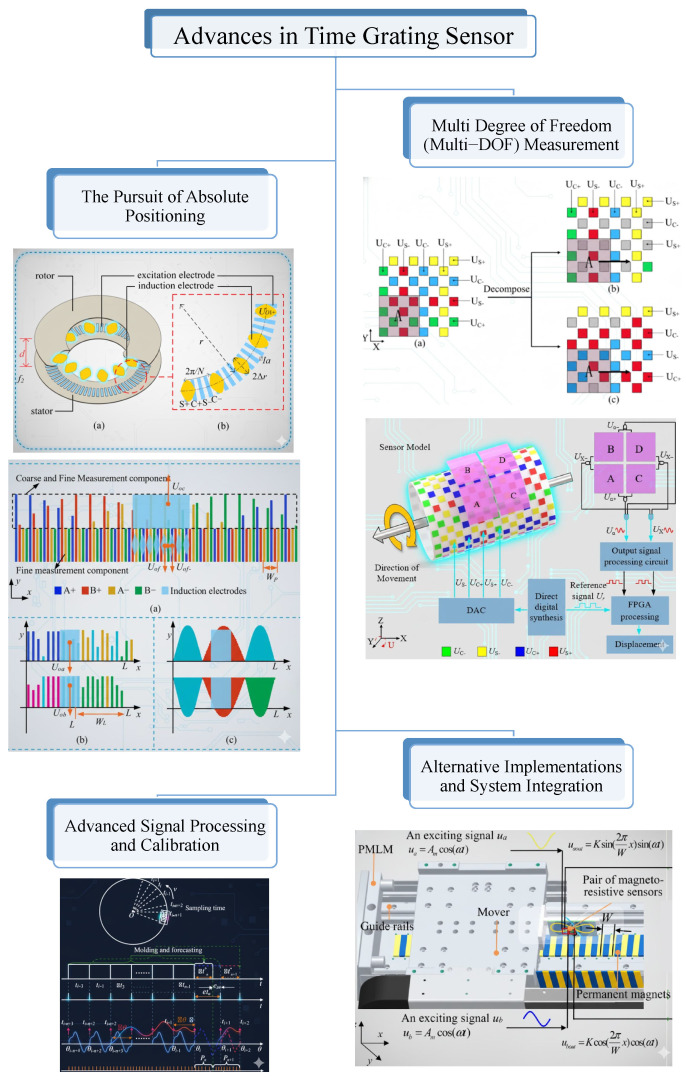
Advances in Time Grating Sensors. The Pursuit of Absolute Positioning, copyright IEEE, reproduced with permission from [62,63]. Multi-Degree-of-Freedom (Multi-DOF) Measurement, copyright IEEE, reproduced with permission from [233,234]. Advanced Signal Processing and Calibration, copyright IEEE, reproduced with permission from [59]. Alternative Implementations and System Integration, copyright IEEE, reproduced with permission from [60]. This figure visually outlines the key research frontiers for Time Grating Sensors, compiling representative concepts for achieving absolute and multi-DOF measurement alongside the essential signal processing and calibration techniques.

Time grating is a newer method in displacement sensing that differs from traditional spatial-domain approaches. Instead of referencing displacement to a physical scale such as an optical wavelength or a grating pitch, the time grating sensor converts displacement into a measurable time interval. As shown in Figure 8, this section reviews its development, from basic concepts to absolute, multi-DOF, and self-calibrating systems.

A critical distinction must be made regarding the metrological traceability of TGS. While a primary advantage of TGS lies in its fabrication—utilizing mature, low-cost processes that avoid precision optical components—the resulting measurement is not intrinsically traceable to the SI definition of the meter (based on the speed of light, *c*). For high-precision applications, the TGS scale requires calibration against a primary standard. This calibration, typically performed ex-situ or in-situ using a co-linear Laser Interferometer (LI) as the reference, establishes the necessary traceability link. Therefore, the TGS’s core advantage is its potential for robust, scalable, and cost-effective interpolation, which relies on an external standard (like LI) to achieve absolute accuracy.

### 6.1. Foundational Principle: The Linear Time Grating Sensor

The core concept of the time grating is to establish a stable, high-speed scanning reference frame—typically a traveling electromagnetic field—that sweeps across a sensor scale. The time difference between the scanner passing a fixed reference point and a moving measurement point is directly proportional to the displacement.

#### 6.1.1. The Basic Concept and Early Demonstrations

The initial work introduced the time grating as a novel capacitive sensor for long-range, nanometer-precision measurement. This was achieved by using orthogonally alternating electric fields as the traveling wave carrier, effectively associating the spatial movement of an object with the phase shift of a time-domain signal. The first prototype demonstrated a remarkable combination of a 200 mm measurement range with an accuracy of ±200 nm and a resolution of 1 nm, establishing it as a promising low-cost technology (Chen Z. et al., 2015, [58]). A subsequent paper elaborated on the physical implementation, which used two rows of differential capacitive sensing electrodes and a sinusoidal grating surface to pick up the displacement signal, further highlighting the low-cost, high-performance potential (Liu X., 2016, [235]).

#### 6.1.2. Overcoming Manufacturing Tolerances and Error Mitigation

A key breakthrough of the time grating principle is its inherent robustness to manufacturing imperfections, a significant advantage over technologies that rely on micro-fabricated physical scales. Research by Peng K. et al. (2017, [236]) revealed that the electric field coupling mechanism provides a strong averaging effect, which suppresses errors arising from the edge roughness of both the sensing and excitation electrodes. This allowed a prototype fabricated with 10 µm-precision PCB technology to achieve a final measurement accuracy of 0.38 µm over a 220 mm stroke—an accuracy far exceeding the manufacturing tolerance. Further analysis identified and mitigated key periodic errors arising from electrical crosstalk and installation misalignment, providing clear design guidelines for optimizing sensor performance (Peng K. et al., 2017, [237]).

### 6.2. The Pursuit of Absolute Positioning

A major thrust of the research has been to advance the technology from incremental to absolute measurement, a critical requirement for many industrial applications. This was primarily achieved by adopting a Vernier-like principle.

#### 6.2.1. The Vernier Principle for Absolute Encoding

The core method for absolute encoding involves using two or more sensor tracks with slightly different spatial periods (e.g., N and N − 1 periods over the measurement range). The phase relationship between these tracks provides a coarse, unambiguous position, while one of the tracks provides the fine incremental measurement.

#### 6.2.2. Absolute Angular Sensors

This Vernier principle was masterfully applied to create high-precision absolute angular sensors. An early design combined three incremental time grating sensors in a multi-stage configuration to achieve absolute measurement with a resolution of ±0.2″ (Yu Z. et al., 2019, [42]). This was followed by a design using two concentric capacitive rings with N and N-1 periods, achieving a raw accuracy of ±2″ over 360° (Pu H. et al., 2019, [238]). The technology was continually refined to create highly compact yet precise absolute angle sensors. A design with a 60 mm outer diameter achieved ±10″ accuracy (Wang H. et al., 2021, [239]), while an even more compact version with a 30 mm outer diameter achieved 12″ accuracy and 0.31″ resolution, demonstrating a powerful solution to the common trade-off between size and precision (Fan X. et al., 2020, [240]). To address the practical challenge of signal routing from the rotor, a cascaded multi-capacitor structure was developed where signals are transmitted between stages via capacitive coupling, eliminating the need for rotor wires and enabling ±2.83″ accuracy (Yu Z. et al., 2024, [62]).

#### 6.2.3. Absolute Linear Sensors and Range Extension

The absolute principle was also applied to linear sensors. A multi-level composite sensor was developed that used the output signals from one stage to excite subsequent stages, achieving an absolute positioning error of ±200 nm over a 200 mm range (Liu X., 2021, [241]). A key innovation for industrial applications was the development of “stitching” techniques to create meter-scale absolute sensors. By designing a 600 mm single-segment Vernier sensor and developing a method to connect multiple segments while eliminating stitching errors, a measurement range of 1140 mm was demonstrated with a consistent absolute accuracy of ±3 µm (Fan X. et al., 2022, [242]). This concept was further refined to create sensors with a 988 mm range and ±3.5 µm accuracy (Peng K. et al., 2023, [61]) and a 1200 mm range with ±5 µm accuracy (Peng K. et al., 2025, [64]), proving the scalability of the technology for large-scale manufacturing equipment. Recent work has also focused on optimizing the coarse measurement component of these absolute systems by using a spatial phase shift method to suppress 4th-order harmonic errors, reducing measurement error by a factor of seven (Pu H. et al., 2025, [243]).

### 6.3. Expansion to Multi-Degree-of-Freedom (Multi-DOF) Measurement

More recently, the time grating principle has been extended to measure multiple DOFs simultaneously, addressing complex motion control challenges.

#### Planar 2D (X-Y) and Cylindrical (Linear + Angular) Sensors

A planar 2D capacitive sensor was developed using a large-area excitation electrode array on a fixed ruler and a small-area sensing array on the moving stage. This design achieved simultaneous X-Y measurement over a 200 × 200 mm area with errors of ±8.2 µm and ±6.8 µm in the x and y directions, respectively (Peng K. et al., 2024, [244]). An L-shaped sensor was also designed to simplify the decoupling of X and Y motion, achieving measurement errors of 17.4 µm and 18.2 µm over a 300 × 300 mm range (Tian Y. et al., 2025, [233]).

In a particularly novel configuration, the principle was adapted to a cylindrical geometry. This sensor, fabricated using flexible PCB technology, is capable of synchronously measuring linear displacement along the cylinder’s axis and the angular displacement around it, effectively solving the Abbe error and installation challenges associated with using two separate sensors (Peng K. et al., 2025, [234]).

### 6.4. Advanced Signal Processing and Calibration

To push the performance limits, significant effort has been invested in signal processing and calibration. An adaptive interpolation method was developed that analyzes the signal’s time period to predict and insert equivalent clock pulses, achieving an interpolation factor of 400× with errors contained within ±1.2″ (Chen Z. et al., 2020, [59]).

Most impressively, a self-calibration methodology was developed to achieve ultra-high accuracy without relying on an external, higher-precision standard. The method uses a dual-sensor system on a dual-bearing turntable, where a multi-stage, interlocked rotation scheme allows the sensors to calibrate each other. By using a concentric, co-axial sensor design, this “correlation constraint relative rotation” method effectively eliminates errors from mechanical misalignment and axis runout, achieving a final calibrated accuracy of ±0.03″ (Zhan B. et al., 2024, [245]).

### 6.5. Alternative Implementations and System Integration

The versatility of the time-domain conversion principle has been demonstrated by applying it to other physical sensing mechanisms. An embedded position detection method was developed for permanent magnet linear motors (PMLM) that uses magnetoresistive sensors to detect the motor’s own periodic magnetic field. This turns the motor itself into a position sensor, achieving a resolution of 0.15 µm in a simple and extremely low-cost implementation (Chen Z. et al., 2021, [60]).

### 6.6. Conclusion and Future Outlook

The research and development of the Time Grating sensor represents a uniquely focused and successful endeavor, systematically evolving a single powerful concept into a broad family of high-performance sensors [246]. The core principle of space-to-time conversion has proven to be remarkably robust, scalable, and versatile. The technology has progressed from demonstrating nanometer-level linear measurement to achieving arc-second level absolute angular positioning in highly compact packages, and more recently to enabling multi-DOF and meter-scale absolute measurements. The demonstrated robustness to manufacturing tolerances and the development of self-calibration techniques position the time grating as a transformative technology with the potential to make high-precision displacement measurement accessible for a wide range of industrial and scientific applications. Future work will likely focus on further enhancing accuracy, expanding to more degrees of freedom, and driving broader industrial adoption.

The research on time grating sensors has gradually expanded a single idea into a family of practical devices [246]. The principle of space-to-time conversion has shown strong robustness, scalability, and flexibility. Early work achieved nanometer-level linear measurements, later extended to arc-second angular measurements in compact setups, and more recently to multi-DOF and meter-scale absolute systems. The technology is tolerant to manufacturing errors and supports self-calibration, which increases its potential for industrial and scientific use. Future work is expected to target higher accuracy, more degrees of freedom, and wider adoption in practical applications.

## 7. Summary and Outlook

This review has analyzed three main technologies in modern precision nanometrology: the laser interferometer, the grating interferometer, and the time grating sensor. Each has its own strengths, limits, and development path, and together they define much of the current metrology landscape. In this final section, we summarize their status and challenges, followed by an outlook on future directions.

### 7.1. Development Status and Technical Challenges

Laser Interferometer (LI): LI is a mature and widely accepted technology, offering unmatched resolution and direct traceability to the SI definition of the meter. Its extensive use in calibration laboratories and ultra-precision manufacturing shows its reliability. The main drawback is its open optical path, which makes it sensitive to turbulence, temperature gradients, and vibrations. This requires complex compensation methods. In addition, optics and demanding alignment limit its miniaturization and make integration into compact systems difficult.

Grating Interferometer (GI): GI provides a practical compromise between high accuracy and industrial robustness. By using a physical grating as a reference, it reduces sensitivity to long optical paths and allows smaller sensor heads. Current research focuses on advanced signal processing for higher resolution, multi-DOF measurement using planar gratings, and architectures tolerant to alignment errors. Challenges remain, especially in grating manufacturing accuracy, pitch uniformity, and line-edge quality, which set a limit on ultimate precision. Careful optical alignment and control of diffraction efficiency are also required to achieve high signal-to-noise ratios.

Time Grating Sensor (TGS): TGS introduces a shift from optical path comparison to space-time conversion. It offers the promise of low-cost, compact, and robust displacement sensing by relying on electronics and signal processing rather than precision optics. Its immunity to many environmental factors is a strong advantage. However, it is still in early development. Challenges include achieving long-range linearity and stability, reducing cross-talk in multi-DOF setups, integrating self-calibration for internal errors, and establishing clear traceability to the SI meter.

### 7.2. Future Directions

Future progress in precision nanometrology will not be led by a single technology but by their integration and complementary use. Several trends can be expected:

1. Hybrid Integration: Combining different sensors to exploit their strengths. For example, using the accuracy of an LI to calibrate arrays of GIs or TGSs can deliver robust and traceable systems.
2. Miniaturization and On-Chip Integration: Embedding sensors into MEMS, robotic tools, or chip-scale systems to enable compact, local feedback and control.
3. Advanced Signal Processing: Using FPGA and AI accelerators for fast compensation of drift, geometry errors, and dynamic effects. Machine learning can help suppress repeatable error sources.
4. Absolute Referencing: Implementing absolute encoding, especially for GIs and TGSs, to remove homing cycles, improve reliability after power loss, and increase safety in critical systems.
5. Standards and Traceability: For TGS and other new methods, developing calibration standards and ensuring traceability to the SI will be essential for acceptance in regulated and scientific applications.

In conclusion, LI will likely remain the reference for highest accuracy, GI will continue as the main choice for integrated industrial systems, and TGS has strong potential where robustness, cost, and scalability are key. Understanding their trade-offs and trajectories will help researchers and engineers design solutions for specific applications and advance toward a new generation of intelligent and widely deployed metrology.

## Figures and Tables

**Table 1:** Comparison of multiple precision nanometrology methods.

	Laser Interferometer	Grating Interferometer	Time Grating Sensor
Accuracy	0.1 nm (vacuum) or 0.9 nm	0.2 nm	100–400 nm
Range	0–10 m	0–2 m	0–0.2 m
Reference	Light source wavelength	Grating period	Time interval
Primary element	High-reflectivity mirror	Precision grating	Coordinate system
Supporting element	Beam splitter and detector	Reference grating	Electronic timing unit
Error sources	Imperfections in optical alignment	Surface irregularities of scale grating	Spatial harmonic components

**Table 2:** Suitability rationale for technologies in key applications.

Application Scenario	Laser Interferometer (LI)	Grating Interferometer (GI)	Time Grating Sensor (TGS)
Semiconductor Lithography	Strengths: Benchmark accuracy; vacuum compatible; traceable toength standard. Limitations: High cost; environmental sensitivity.	Strengths: High accuracy; excellent for planar X-Y stages. Limitations: Grating errors (stitching);ess vacuum heritage.	Strengths: N/A (low maturity). Limitations: Unproven accuracy/stability at picometerevel.
Precision Machine Tools	Strengths: Highest possible accuracy for calibration. Limitations: Very sensitive to shop-floor environment (vibrations, thermal).	Strengths: Industry standard; robust and sealed; good cost-performance. Limitations: Accuracyimited by scale quality.	Strengths: Excellent robustness; high immunity to vibration/contamination. Limitations: Newer technology;ong-term reliability data pending.
Compact Embedded Modules	Strengths: N/A. Limitations: Inherently bulky form factor; requires clear optical path.	Strengths: Compact readheads available; flexible integration. Limitations: Still requires a physical scale.	Strengths: Potentially very compact sensor head; simple mechanical interface. Limitations: Internal scanner adds some complexity.
Cost-Sensitive Industrial	Strengths: N/A. Limitations: Prohibitively high cost for mass applications.	Strengths: Mature technology with wide range of cost points. Limitations: High-performance versions are still costly.	Strengths: Projectedow cost by replacing nano-fabrication with electronics. Limitations: Economy of scale not yet fully realized.

## Data Availability

The data presented in this study are available on request from the corresponding author.
